# Expanded forehead flap in Asian nasal reconstruction

**DOI:** 10.1038/s41598-023-30245-3

**Published:** 2023-04-04

**Authors:** Muqian Wei, Xi Bu, Guanhuier Wang, Yonghuan Zhen, Xin Yang, Dong Li, Yang An

**Affiliations:** Department of Plastic Surgery, Peking University Third Hospital, 49 North Garden Road, Haidian District, Beijing, 100191 China

**Keywords:** Diseases, Medical research

## Abstract

This article reviewed our experience of Chinese nasal reconstruction over 12 years and evaluated the effect of expanded forehead flap both aesthetically and functionally. The special skin type and other anatomic features of Chinese patients was understood thoroughly during the treatment. This article thus catered for the need of multiracial nasal reconstruction. We analyzed existing clinical data and demonstrated a typical case in detail. The postoperative result supported our strategy which advocated the extensive application of expanded forehead flap, together with flip scar flap as the internal lining. The features of Chinese patients also prompted the use of costal and auricular cartilage. Emerging technology like 3D-printing would benefit nasal reconstruction from more aspects.

## Introduction

The reconstruction of nose remains one of the most challenging procedures in plastic surgery. Oncologic resection, trauma, infection, and congenital conditions are the most common causes of nasal defects. Restoration of aesthetic and physiological functions are both mandatory. Sushruta first described the nasal reconstruction surgery in his treatise *Sushruta Samhita*^1,2^. The technique is practiced almost unchanged to this day, and thus the pedicled forehead flap is known as the Indian flap or Indian method^3^.

After years of progression, the principle of nasal reconstruction hasn’t changed much, which was concluded as replacing three layers: the internal lining, structure, and outer skin envelope^4^. The appearance and blood supply of the forehead are generally ideal for nasal reconstruction. However, Chinese patients have special characteristics to be considered by: their skin is type III to IV Fitzpatrick’s classification and tends to present postinflammatory hyperpigmentation significantly^5^. It’s also vulnerable to excessive scar formation after closure of the donor site^6^. The expanded forehead flap was thus considered as the first choice for nasal reconstruction by Lu et al.^7^ Other techniques include grafts and nasolabial flap. A composite graft or a folded nasolabial flap is suitable for small and medium defects^8^. Related reports of nasal reconstruction for Chinese patients are still insufficient and the optimal strategy remains elusive. Here, we would like to share our experiences on nasal reconstruction for the period spanning 2010–2021. The goal of this article is to present the currently preferred methods in Chinese nasal reconstruction and evaluate the application of expanded forehead flap comprehensively.

## Patients and methods

**Figure 1:**
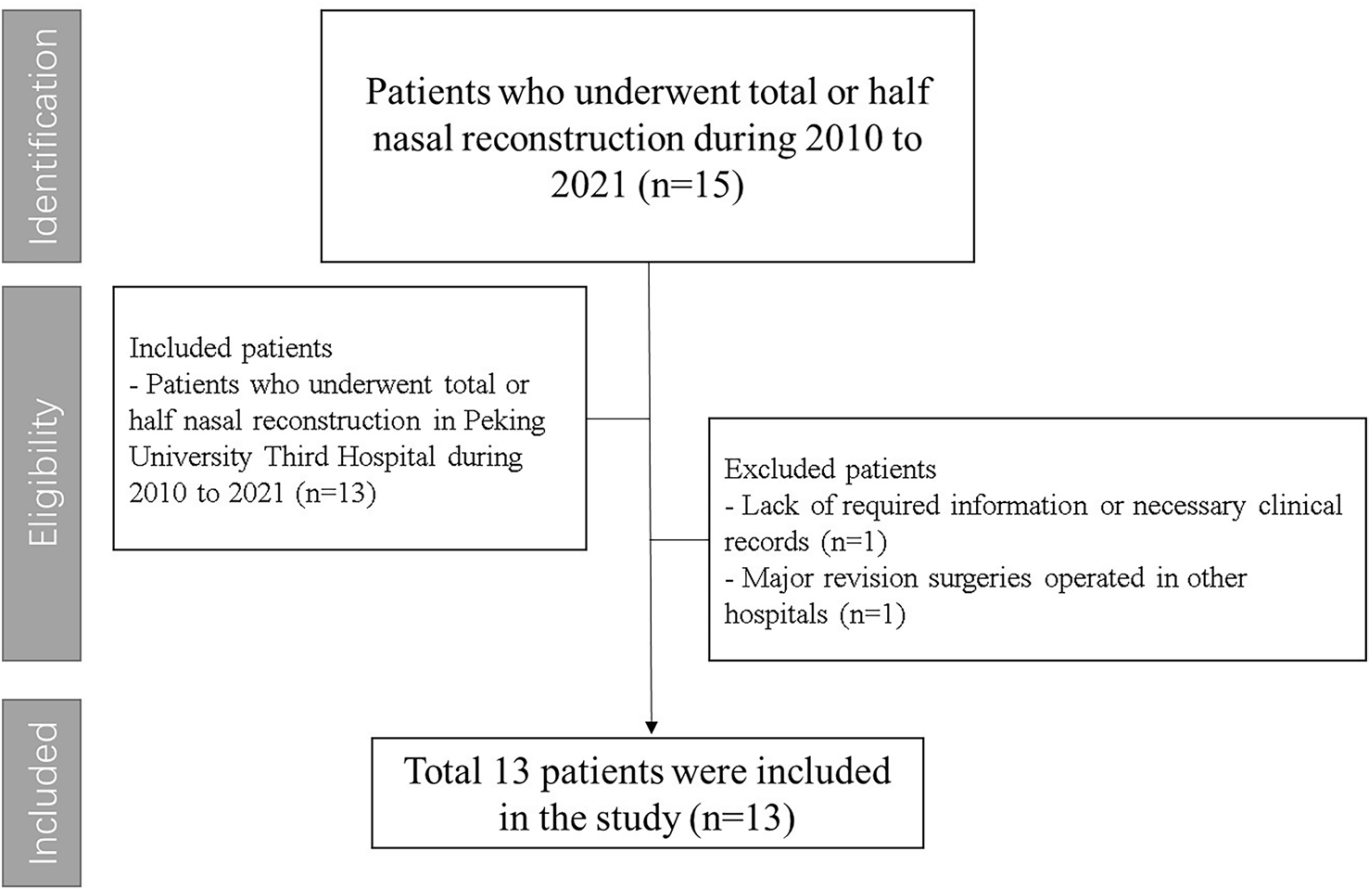
Inclusion and exclusion process of patients in this study.

We performed a retrospective study on patients who underwent nasal reconstruction in Peking University Third Hospital from 2010 to 2021. Patients were included in this study if they had undergone nasal reconstruction in this period. Two of them were excluded according to our exclusion criteria (Fig. 1).

The following documents of every patient were reviewed from the hospital’s database: operative recordings, operating room nursing records, anesthesia notes, and medical records. Patients’ demographics were collected and listed.

All procedures were performed according to the hospital’s established protocols under general anesthesia. Respective clinical decisions for the reconstruction of internal lining, structure and outer skin envelope were extracted from available materials, together with relevant treating information. A preliminary operation was adopted to implant a tissue expander as a stage of forehead flap reconstruction, and the usage was documented.

### Preoperative design

**Figure 2:**
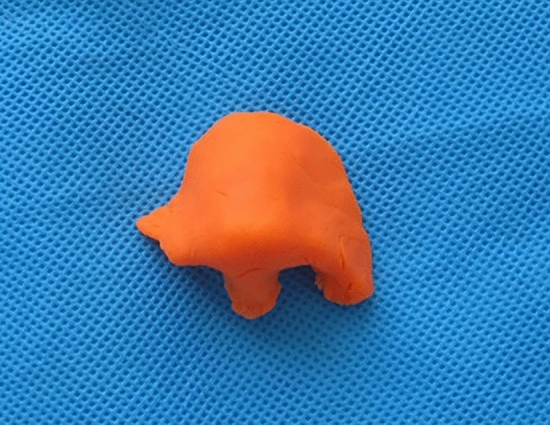
A clay model of the preoperatively designed nose.

Total or half nasal reconstruction was decided and performed according to the defects of nasal subunits. Preoperative images of the patient were taken, and standardized 3D photographs (VECTRA XT Imaging System; Canfield Scientific, Parsippany, NJ, USA) were used to design an ideal nasal shape through consultation with the patient. (VECTRA is a commercially available system that uses facial photographs to visualize the face in 3D.) A nasal model was then made by hand with clay according to the ideal 3D shape (Fig. 2). The flap size was also determined according to measured parameters of nasal aesthetic subunits: the length, height and radian of nasal dorsum, the protrusion of nasal tip, the columellar-labial angle, the nasofrontal angle, and so forth. Finally, the median forehead flap or paramedian forehead flap was selected according to the height of patient’s hairline.

### Surgical techniques

The procedure was divided into at least three steps.

#### Stage I: implantation of tissue expander

**Figure 3:**
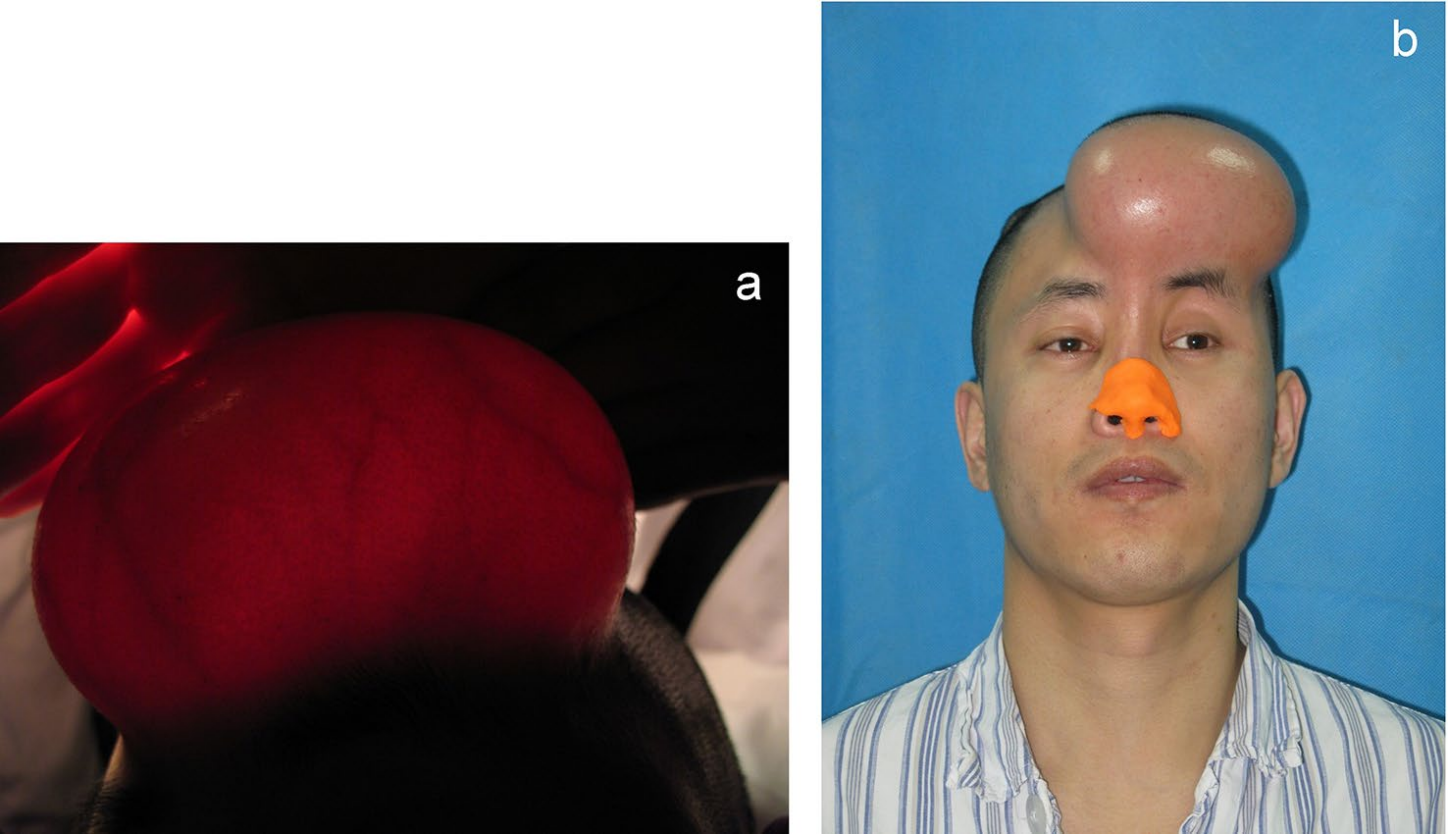
Patient with the tissue expander inserted in forehead. (**a**) The trend and distribution of vessels in the expanded forehead skin. (**b**) Maximal expansion of forehead skin.

A surgical incision about 4 cm in length was made 2–3 cm above the hairline. The skin, subcutaneous and galea layers were separated in turn. Lower bound of the implantation lacuna was bluntly dissected from periosteum to the level of upper eyebrow margin and 1–2 cm above the anterior hairline. The volume of expanders varied from 180 to 240 ml according to the actual situation and the selected cylindrical expander was placed in the lacuna subgaleally. A negative pressure suction tube was placed and the wound was sutured in layers. Saline 15 to 20 ml was injected into the expander in advance. Postoperative injection was performed every week until required volume was achieved (Fig. 3).

#### Stage II: extraction of tissue expander and forehead flap transfer (total/half) nose reconstruction

**Figure 4:**
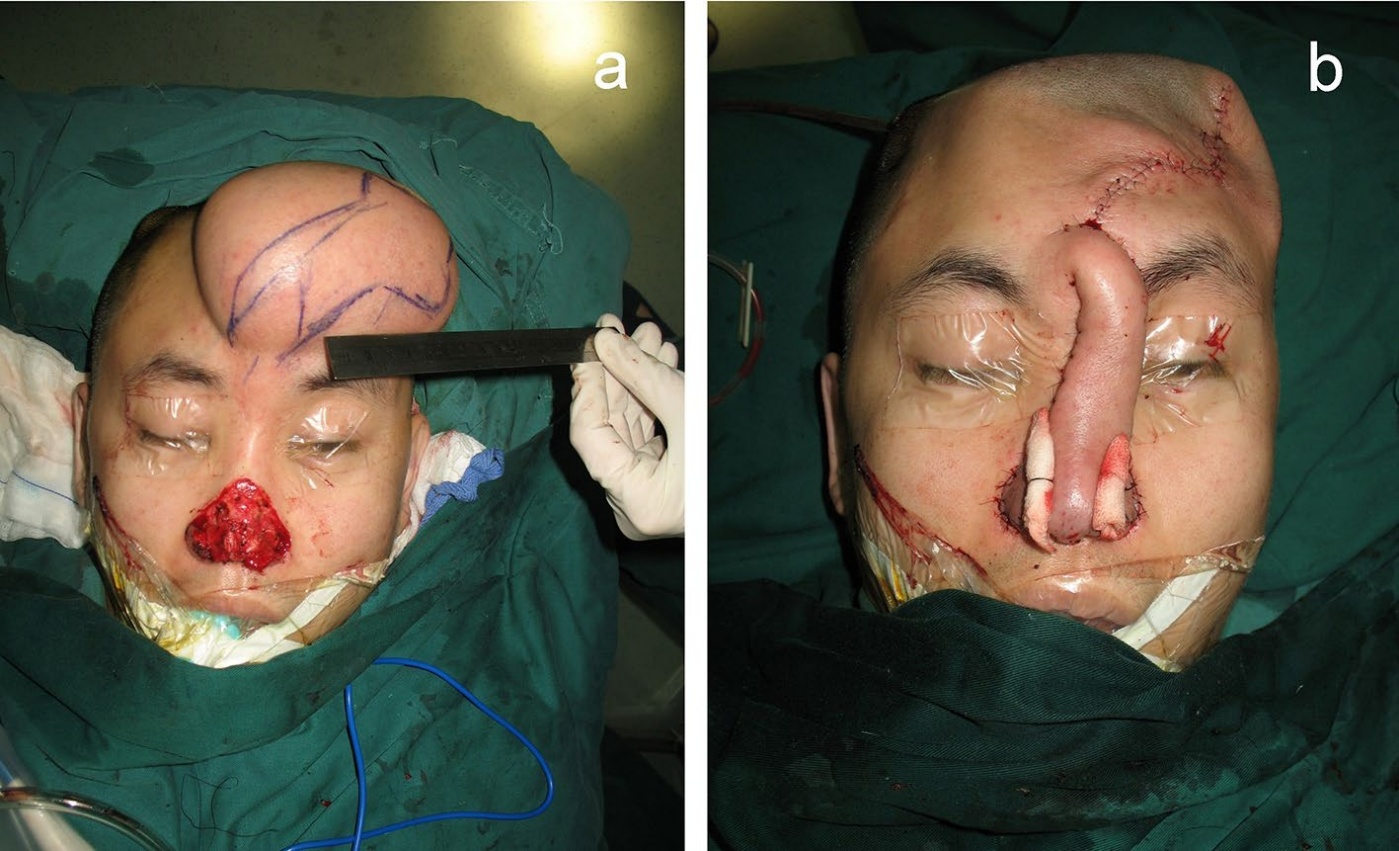
(**a**) Preoperative design of the expanded forehead flap. (**b**) Intraoperative photograph of the reconstructed nose and the primarily closed donor site (same patient in Fig. 3).

The contour of the reconstructed nose was determined according to the objective parameters of aesthetic subunits measured preoperatively and enlarged by 1 to 1.5 cm. The three-lobule forehead flap was designed with one-side supratrochlear or supraorbital vascular bundle as the pedicle. The nasal defect was replaced satisfactorily with the transposed simulated cloth-like flap. Costal and/or auricular cartilage can be harvested according to the size and shape of the alar/septal cartilage which required reconstruction. The flap was cut from the distal end through the capsule tissue of the expander, and stripped bluntly to the location of glabella and the pedicle gradually, without injuring the supratrochlear vascular bundle. The lining was usually reconstructed with residual skin around the nasal defect, which is dissected and turned over to replace internal lining tissue. Cartilage grafts was sculptured and fixed to rebuild the nasal support. Distal end of the three-lobule flap was thinned and turned inward to be sutured with the foregoing reconstructed lining. The two bilateral lobules of the flap designed as ala were turned towards the subcutaneous tissue to elevate nasal tip. It is important that when the expanded forehead flap was transferred to the nose, there should be no tension at the pedicle. After dissection of the flap, its median end and the dissected upper lip were turned and sutured at the fixed point of the columella, forming the bilateral wings of the columellar flap, fixed at the fixed point of the nasal ala. These three fixed points were vital for nasal reconstruction and must be accurate. Subsequently, the part of the flap forming the two nostrils and the nasal lining were sutured together, respectively. The lining could be slightly cut short when it was too long, to ensure that the distal end of the left and right lobules were able to turn inward and formed the ala. The distal three lobules of the flap were respectively folded into nasal columella and ala. The donor site and pedicle tube were sutured directly (Fig. 4).

#### Stage III: pedicle restoration

**Figure 5:**
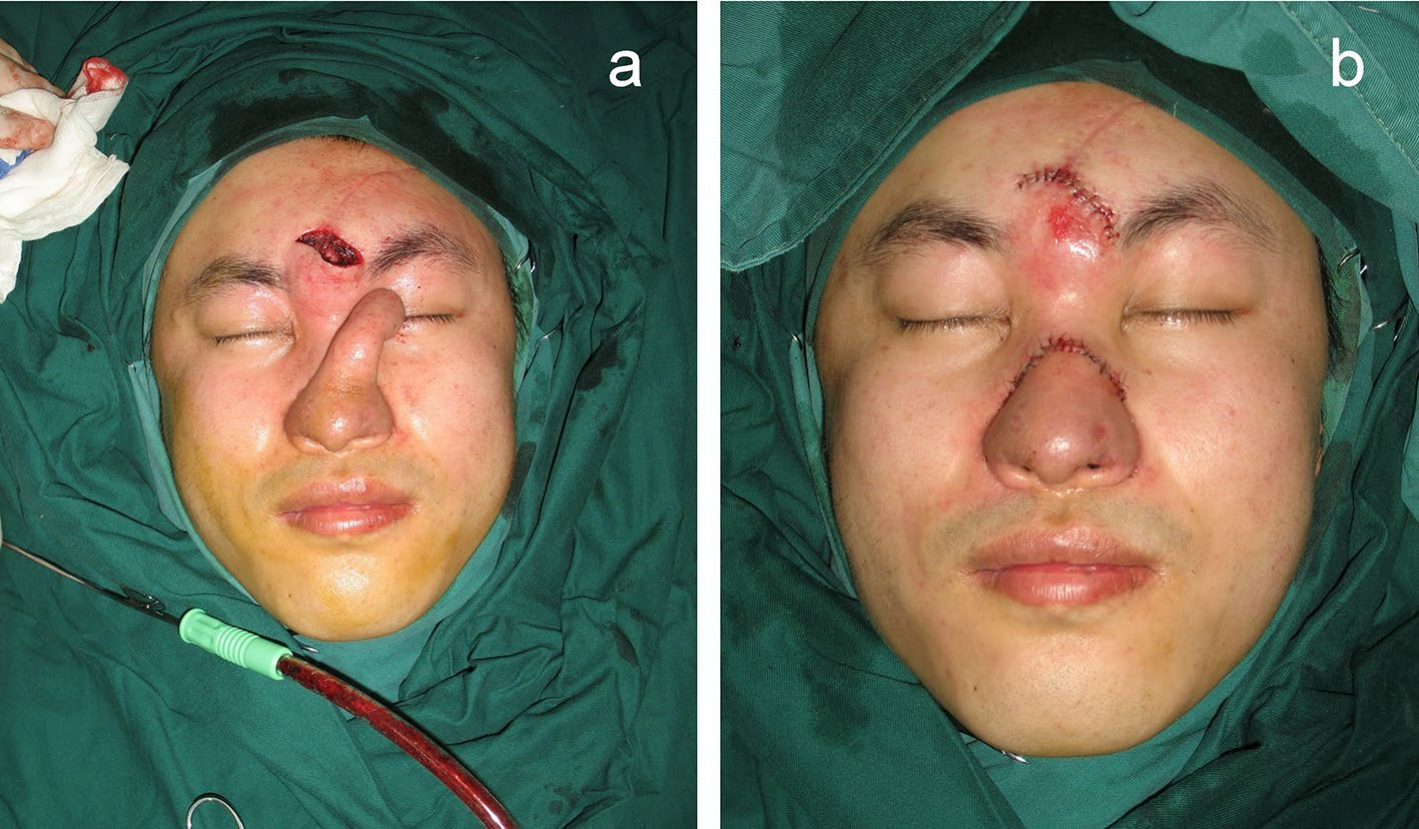
(**a**) Resection of the pedicle. (**b**) Restoration and closure of the defect (same patient in Fig. 3).

The pedicle of the flap was resected and restored 3 weeks after Stage II. The flap was transected along the pedicle. The eyebrows on both sides were adjusted to maintain symmetry, and the portion of the nose in poor shape was trimmed. Silicone tubes with gauze were inserted into the nostril for 10 days. Then they were replaced with silicone rubber nostril maintainers of appropriate size. The silicone maintainers were worn intermittently for at least 6 months. We suggested wearing the maintainers as long as possible to avoid injuries of nasal mucosa. Timing of taking off nostril maintainers were related to patients’ compliance and their social needs in daily living. The nostrils and nostril maintainers were cleaned daily using sterile physiological saline (Fig. 5).

### Postoperative evaluation

The postoperative outcome during the healing phase was accepted well by all patients and no post-surgical dissatisfaction occurred. Patients reconstructed with expanded forehead flap were followed and assessed with questionnaires from both an esthetic and a functional perspective. The questionnaire consists of 2 parts: (1) the Chinese Nasal Obstruction Symptom Evaluation (NOSE) questionnaire translated and validated by Chinese researches already^9,10^; (2) the Chinese Rhinoplasty Outcome Evaluation (ROE) questionnaire translated and validated by Chinese researches^11^. All questionnaires were filled out by patients under the guidance of professional doctors. Internal consistency was measured with Cronbach’s alpha. Cronbach’s alpha > 0.70 indicated a good consistency.

### Ethics approval and informed consent

All methods in this study were performed in accordance with the guidelines and standards of the institution. The study was approved by the Ethics Committee Office of Peking University Third Hospital. The informed consent was obtained from patients involved in the study including the publication of information/image(s) in an online open access publication.

## Results

13 patients were included with 8 male, 5 female and the mean age was 45 years (range 26–70 years). Most patients were Han people (10 of 13). Main causes of nasal defects were trauma (5 of 13) and cancer (3 BCC; 1 SCC), others were 2 nasal contracture deformities, 1 mucormycosis and 1 congenital deformity. The average BMI was 22.78 (range 17.58–32.05). Ala (10 of 13) is the most affected site in our cohort (Supplementary Table S1).

**Figure 6:**
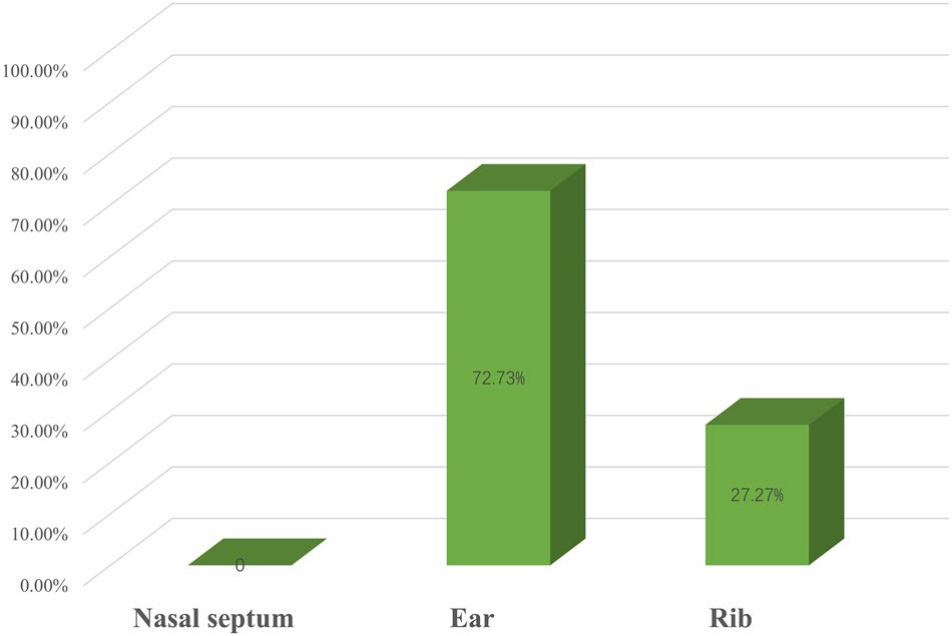
Patients’ donor sites of cartilage grafts.

7 patients underwent total nasal reconstruction and the other 6 patients received half nasal reconstruction. There were no serious perioperative or postoperative complications reported. Survival of the flap was 100% and no ischemia occurred even for 2 patients who actively smoked (1 used the expanded forehead flap, 1 used the nasolabial flap). Total nasal reconstructions were all performed using a 3-stage expanded forehead flap. The tissue expanders were rectangular or reniform, and the volume ranged from 50 to 150 ml, depending on the expected thickness of the expanded forehead flap and the characteristics of certain patients. 2 cases of leakage occurred after the primary implantation of tissue expander and we replaced them with more suitable ones in a secondary operation. Ipsilateral nasolabial flap, composite graft and full-thickness skin graft were added to the surgeon’s arsenal as complementary choices for half nasal reconstruction (1 used nasolabial flap for lining reconstruction, 1used nasolabial flap alone, 1 used nasolabial flap together with a full-thickness skin graft, 1 used auricular composite graft alone) when the size of patient’s defect is more limited. As for the flap design, 6 out of 10 expanded forehead flaps were based on contralateral vascular pedicle (Parkland design) and the other 4 were based on ipsilateral pedicle (axial design). Nasolabial flaps were all ipsilaterally designed. 10 patients received cartilage graft transplants to get better structural support. We only use cartilage harvested from ear and rib: 8 patients used ear cartilage graft and 3 patients used costal cartilage, with 1 patient used both (Fig. 6).

The inner lining was reconstructed with flip scar flaps (9 of 13), nasolabial flap (1 of 13), radial forearm free flap (1 of 13) and full-thickness skin graft (1 of 13) when needed. Alloplastic material was only utilized in 1 patient for nasal dorsum augmentation out of specific aesthetic consideration. The average time of pedicle division was 8.36 weeks (ranging from 2 to 24 weeks), with some patients delayed because of allopatric or some other practical issues. Revisions were needed when the patient’s condition was generally poor or the defects involved not only the nasal structure but other facial components. 5 patients required revisions in total. 2 of them received 7 and 4 revisions respectively after total nasal reconstruction for the defects were particularly large and severe. Personal aesthetic demand and practical limitations (e.g., patient’s work schedule) also accounted for the multiple revisions (Table 1).

**Table 1:** Surgical procedures.

Patient	Total/half nasal reconstruction	Preoperatively implanted expander and its usage	Reconstruction for internal lining	Reconstruction for structure	Reconstruction for skin envelope	Flap design	Time to pedicle division	Use/type of alloplastic materials	Revision
1	Total	Yes, Xinsheng silicone rubber soft tissue expander (150 ml, rectangle; saline injection: 330 ml)	Flip scar flap	Bilateral conchal cartilage	Expanded forehead flap	Parkland design	2 weeks	No	No
2	Total	Yes, Xinsheng silicone rubber soft tissue expander (150 ml, rectangle; saline injection: 302 ml)	Flip scar flap	Costal cartilage	Expanded forehead flap	Axial design	15 weeks	No	7
3	Half	Yes, reniform expander (150 ml)	Flip scar flap	Ipsilateral conchal cartilage	Expanded forehead flap	Parkland design	5 weeks	No	3
4	Total	Yes, silicone rubber expander (150 ml)	Left RFFF	Costal and conchal cartilage	Expanded forehead flap	Parkland design	5 weeks	No	4
5	Total	Yes, silicone rubber expander (150 ml)	Flip scar flap	Bilateral conchal cartilage	Expanded forehead flap	Parkland design	7 weeks	No	No
6	Half	Yes, silicone rubber expander (150 ml, rectangle)	Ipsilateral nasolabial flap	Contralateral conchal cartilage	Expanded forehead flap	Axial design	6 weeks	No	No
7	Half	Yes	Flip scar flap	–	Expanded forehead flap	Parkland design	3 weeks	No	No
8	Total	Yes, reniform expander (50 ml) at right 1/3 forehead; silicone rubber expander (150 ml, rectangle) at left 2/3 forehead	Flip scar flap	Right conchal cartilage	Expanded forehead flap	Axial design	24 weeks	No	No
9	Total	Yes, expander (100 ml)	Flip scar flap	Bilateral conchal cartilage	Expanded forehead flap	Axial design	5 weeks	Expanded polytetrafluoroethylene	2
10	Total	Yes, expander (80 ml), switched to expander (100 ml, rectangle) after 3 months	No defect	Costal cartilage	Expanded forehead flap	Parkland design	4 weeks	No	No
11	Half	No	Ipsilateral flip scar flap	–	Ipsilateral nasolabial flap, FTSG	–	16 weeks	No	1
12	Half	No	Ipsilateral flip scar flap	Ipsilateral conchal composite graft	Ipsilateral conchal composite graft	–	No	No	No
13	Half	No	Ipsilateral folded nasolabial flap sheared as FTSG	–	Ipsilateral folded nasolabial flap	–	No	No	No

*RFFF* radial forearm free flap, *FTSG* full-thickness skin graft.

Postoperative recovery and appearance of the reconstructed nose were satisfactory. Both parts of the questionnaire showed strong internal consistency reliability, with Cronbach’s alpha 0.938 ((1) NOSE) and 0.889 ((2) ROE). Most patients did not report any degree of nasal stuffiness in NOSE, except for one case of mild discomfort (Patient 4). The median ROE score was 22. The evaluation generally shows an excellent result after total or half nasal reconstruction with expanded forehead flap (Table 2).

**Table 2:** Postoperative evaluaion on NOSE and ROE scores in patients reconstructed with expanded forehead flap.

Patient	NOSE score	ROE score
1	0	22
2	0	24
3	0	24
4	20	18
5	0	19
6	0	22
7	0	24
8	0	21
9	0	24
10	0	20
MEDIAN	0	22

*NOSE* nasal obstruction symptom evaluation, *ROE* rhinoplasty outcome evaluation, *SD* standard deviation.

## Case presentation

### Case 1: 36-year-old woman with mucormycosis of the nose

A cavity was present in the face owing to disappearance of the nasal contour together with cartilage and nasal bone after the management of mucormycosis in an outside hospital. The cavity reached the oral cavity and the uvula disappeared. Rhino-orbital-cerebral infection is the most common presentation of the invasive fungal disease, involving adjacent nasal anatomy, sinuses, palate, and orbit^12,13^. Specialist examination revealed partial eyelid adhesions (ankyloblepharon), facial scars from the eyelid to the upper lip, and complete disappearance of nasal structure. The treatment plan was delicately designed (Supplementary Fig. S2). A 150 ml cylindrical tissue expander was inserted below the frontal galea first (Supplementary Fig. S3). Saline injection was slowly administered to 400 ml in 3 months after the implantation (Supplementary Fig. S4). Three months later, a left radial forearm free flap (11cmx7.5 cm) was transferred to the nose to fill the nasal cavity. A 12 cm long right great saphenous vein was cut as a bypass graft, and the radial artery and cephalic vein were anastomosed with the facial artery and vein (Supplementary Fig. S5). The flap was transferred to the nose, folded in half, and secured with interrupted sutures (Supplementary Fig. S5). Two months after the free flap transplantation, the right 7th rib and costal cartilage were harvested to reconstruct nasal bone and nasal tip-columella strut, respectively (Supplementary Fig. S6). Two months after the surgery, the skin and some subcutaneous fats of the free flap were removed. Total nasal reconstruction was performed with expanded paramedian forehead flap, and a 2 cm × 6 mm auricular cartilage was cut from both sides as alar struts. The forearm free flap was then trimmed to form internal lining, and the extended forehead flap as outer skin envelope (Supplementary Fig. S7). The pedicle of the forehead flap was dissected after a month (Supplementary Fig. S8). Flap revision and dermabrasion was performed three times postoperatively to obtain optimal result. In the later follow-up, the color and texture of the reconstructed nose were similar to that of adjacent skin, and the incision scar at the edge of the flap was occult (Supplementary Fig. S9). The patient was very satisfied with the result.

## Discussion

In order to obtain a coordinated result, the subunit principle in rhinoplasty was proposed by Burget and Menick^14^. It was concluded that if more than 50% of one aesthetic subunit was involved, then the optimal choice would be removing the leftover and reconstruct the whole subunit, including all three lamellae^15^. For the same reason, total or subtotal nasal reconstruction is considered when the defect is large and encompasses two or more nasal subunits.

The paramedian forehead flap provides esthetically satisfying skin color and texture to match surrounding tissue. It is well-vascularized and could be applied to smokers, considering that the blood supply of forehead tissue derived from the supratrochlear artery and other collateral arteries, which compose a complex vascular network with abundant nourishment^16,17^. For this reason, the paramedian forehead flap was put forward by Millard as a two-stage procedure based on unilateral vascular pedicle to obtain a more effective length, smaller rotation angle and more applicable pedicle^18–20^. The forehead flap surgery is generally safe to perform and anticoagulation therapy is not needed^21^. For these reasons, it has been the first choice for nasal reconstruction so far.

The remaining problem is that forehead tissue contains 4 layers: skin, subcutaneous fat, the frontalis muscle and a thin layer of areolar tissue, which makes all forehead flaps thicker than normal nasal tissue and hard to form an ideal 3D shape^22,23^. Menick^22,24^ preferred to perform a modified 3-stage forehead flap to overcome this problem. However, it may not be suitable for East Asian patients because of racial characteristics. Different from Caucasians, the skin of East Asians mainly pertains to Fitzpatrick skin type III to IV with a higher risk of keloids and hypertrophic scars^6,25–27^. It is thicker with dense ligamentous attachments, greater amount of melanin and contains more collagen, which results in a higher tendency to develop hyperpigmentation and scar formation^5,28,29^. Hsiao et al^6^ therefore advocated some refinements based on the subunit principles in nasal reconstruction for ethnic Asian patients. Radovan^30^ first presented his technique of chest skin expansion at the Annual Meeting of American Society of Plastic and Reconstructive Surgeons. Not long after that, this technique was applied to nasal reconstruction and repairment of other units on the head for apparent advantages^31–34^. The application of tissue expansion efficiently helps to solve this problem as the donor site can be totally free of tension and easy to close primarily, creating a minimum scar line following this modality^23^. It can also thin the terminal branch of the flap to 1.2–2 mm so that the flap would perfectly match native nasal skin and might be folded to reconstruct inner lining or perform some other manipulations^35^. Therefore, tissue expansion of the forehead flap provides abundant bulk of tissue for reconstruction, which is also supple, well-vascularized, easy to operate on and donor-site protective, especially for Chinese or East Asian patients.

Here, we extensively used expanded paramedian forehead flap in all total nasal reconstruction and 50% of half nasal reconstruction out of its attractive advantages and special consideration for Chinese patients. Smaller defects could also be repaired with other flaps (e.g., nasolabial flap) and skin grafts in accordance with patients’ demand. The expanded forehead flap was categorized by Wang et al.^36^ as Type II flap and the expander was suggested to be inserted submuscularly for safety concerns. The volume of the expanders we used was mostly 150 ml. The volume of fluid injection was approximately twice the volume of the expander. Expansion often proceeded once a week, with 30–40 ml fluid injected each time. Subsequent surgery was performed about 1 month after the final expansion to help stabilize forehead tissue. There is no need to dissect the pedicle after elevation of the flap because it is long enough and the size of the flap can be rather adaptive^36^. Lu et al.^7^ agreed with the priority of expanded forehead flap when dealing with non-Caucasian patients’ defects in consideration of hairline and the tendency to scar, which was in accord with our view. Pinto et al.^37^ treasured the expanded forehead flap as well and claimed that it was their gold standard to provide external skin coverage in nasal reconstructions. Limitations of this technique include a prolonged surgical procedure, inconvenience in daily life and an embarrassing appearance with the expander implanted inside. However, the awkward situation could be evaded well through careful negotiation and planning with patients prior to the expansion procedure. From our experience, we did not receive report of dissatisfaction concerning this problem when preoperative consensus was reached. According to other researches, patients may also encounter kinds of problems or complications, such as infection, extrusion, wound dehiscence and ruptures^38^. The prevention of infection could be managed with an algorithm put forward by Dong et al.^39^. Nevertheless, we did not observe any complications directly related to the transferred flap, like necrosis or infection, except for two cases of leakage during the gradual process of tissue expansion. Radwan et al.^40^ attributed the occurrence of leakage to 3 reasons: (1) inadvertent puncture of the tubing or the prosthesis; (2) improper securing of the connector; (3) use of an appropriate needle into the injection port. Thus, it was recommended that the tubing be trimmed, the connector be well protected and injections be completed at varied locations with a small needle. Our experience suggested that replacing the leaky expander with a more suitable one in time could figure out this problem effectively without any sequelae and do no harm to the final result. The forehead has been argued as one of the expander locations with the lowest incidence of overall complications, hence the extra injury that might occur to patients treated by expanded forehead flap is minimized^38,41^.

The racial features of Asian patients not only highlight the use of expanded forehead flap, but are also concerned with the donor site selection of cartilage grafts needed for structural support. In our series, 10 of 13 needed cartilage grafts for structural reconstruction, which provide reliable support to prevent the inner lining and outer envelope from collapsing especially for full-thickness defects. Donor sites include the ear and costal region. Cartilage from the remnant septum has been taken as another important source of grafts by doctors in view of its outstanding physical properties and harvesting convenience^4,42–44^, while we recognized it as an inappropriate choice for Chinese patients. Unlike Caucasians, the septal cartilage of Asian people is weak and scarce, especially when an extensive defect affects the cartilaginous septum, which necessitates the use of the auricular and costal cartilage^45,46^. In our series, auricular cartilage was mainly used for ala, nasal tip and columellar reconstruction. Costal cartilage was designed as a three-dimensional strut to support the nasal tip, columellar, ala or dorsum and reinforce the nasal septum. It was as well treasured by our Chinese counterparts because the costal cartilage was satisfying in both quantity and quality, particularly when a large cartilaginous defect occurred^7,47^. The intrinsic curve and suppleness of conchal cartilage make it the first choice to design alar batten grafts or replace lateral cartilage, while the prominent stiffness and abundance of costal cartilage are ideal to play the role as a central support element or dorsal onlay graft^48–50^. Sannier et al.^51^ also preferred to use ipsilateral conchal bowl cartilage and suggested the exploitation of costochondral cartilage when cartilage was in great demand.

As one of the most challenging tasks, reconstruction of the internal lining could be realized in various approaches^52^. The application of different techniques, from the simplest split or full-thickness skin grafts to mucosal flaps, pedicled flaps and free flaps, are reviewed and concisely summarized by Philips^4^ along with their pros and cons. Unlike the viewpoint of Philips considering the turn-in pedicled flap far from being ideal, Thornton^51^ preferred the usage of forehead flap skin for turn-in lining, which corresponds with the experience of Noel et al.^53^ Weber and Wang^54^ proposed the reconstructive ladder and several principles in nasal-lining reconstruction to stratify reconstructive modalities from the least (secondary intention) to the most complex (free-tissue transfer). Flip scar flap, also known as hinge-over flap, is a turndown flap of the released scar^55^ and has been our first-place choice for the reconstruction of internal nasal lining according to the senior author’s experience. RFFF and nasolabial flap were also used when the residual skin tissue is not enough or not suitable. We recognized that mucosa tissue is the optimal choice for airway humidification, while the surgeons were not accustomed to endoscopic operation to replace nasal lining with mucosa. From our view, the use of flip scar flap did not result in any adverse consequence, as the postoperative evaluation showed. The selection of the internal lining is similar to that of our Chinese counterpart^56^. Zenga^57^ argued that the hinge flaps had the greatest reach and vascularity, but they required a prolonged period of time between injury and repair. From our perspective, the average time needed to heal is generally acceptable. The use of titanium mesh for nonradiated patients with an extensively large mucosal defect was also approved by scholars, although we did not take this technique into account^57–59^.

The NOSE and ROE scores are commonly used to evaluate the outcome of rhinoplasty both aesthetically and functionally. In our series, the scores of both parts in the questionnaire were satisfying^11,60^. The NOSE score mainly focus on the severity of nasal obstruction, and the ROE score includes 6 questions, measuring comprehensive attitudes of patients towards rhinoplasties. Our choice to use flip scar flap did not occlude the airway as some may suggest, and thus increase patients’ quality of life. The evaluation supports the extensive use of expanded forehead flap, together with flip scar flap to form internal lining.

As regards the continuous progress achieved in the area of rhinoplasty, advances in science and digital technology have benefited the nasal reconstruction surgery a lot. The application of 3D preoperative planning and templating in forehead flap nasal reconstruction has already been reported. Zeng et al.^61^ scanned the patient’s 3D facial model including the nasal defect and got a normal 3D model with the highest similarity matched in a database. The normal model found by a personalized algorithm was then transformed into a 2D flattened one to help determine the scope and usage of the forehead flap, which is more reliable and precise than the conventional empirical approach depending on the surgeon’s personal experience and opinions only. Fishman et al.^62^ also utilized a conformal “unwrap” tool to obtain an ideal 2D skin flap shape from the patient’s flattened 3D nasal geometry. The following printed layout could therefore be referenced and traced on the forehead for intraoperative use. Further studies are still needed to modify the flattening algorithm, improve the operating steps, and confirm the subjective and objective outcome of this computer-assisted design and manufacturing pipeline so that it could be applied more widely in nasal reconstruction. Moreover, 3D-printing technology can fabricate a 3D surgical template of the complex structure of patient’s normal nose, to guide the reconstruction visually^63,64^. Combined with the promising bio-engineered materials, 3D printing has also been explored and applied in the field of osteogenesis and chondrogenesis, like the regeneration of nasal and auricular cartilage, and its feasibility in regenerative medicine was demonstrated by trials^65–68^. The intricate anatomical structure of nose calls for the help of 3D printing together with bio-engineered materials to satisfy patients’ need for personalized medicine better. Taking the dynamic aesthetic into consideration may be another vital direction for improvement once capable technical assistance becomes available.

This study had several limitations. First, it is a retrospective study, and the sample size was constrained. It is also subject to limitations associated with retrospective design. The impact of COVID-19 pandemic prevented us from recalling patients distributed countrywide, to take pictures and conduct furtherer objective assessments by professional doctors.

## Conclusion

Nasal reconstruction is a complex, historic and artistic work. With an increasing possibility of treating racial/ethnically diverse patients, this article reviews our experience to treat Chinese patients over a 12-year period and provides an important reference for an improved race-based treatment. The expanded forehead flap is therefore proven to be a versatile technique for nasal reconstruction. From our perspective, it is deemed as the golden standard for Chinese nasal reconstruction, with some other techniques being acceptable alternatives under specific circumstances. Costal and ear cartilage are important to stabilize the structure and prevent scar contracture. The flip scar flap is also an effective choice to reconstruct internal lining. Going forward, modest progress has been realized in the field of interdisciplinary research: big data and digital technology have already benefited nasal reconstruction in terms of flap design. Furthermore, it sees that 3D printing technology and bio-engineering materials as ingredients would collectively offer outstanding new choices and opportunities to a wider range of patients in the future, holding promise for improving the access and quality of nasal reconstruction.

## Supplementary Information

Supplementary Information.

## Data Availability

Data used or analyzed during this study are included in the article. Detailed datasets are available from the corresponding author on reasonable request.
