# Supplementary material for: Expanded forehead flap in Asian nasal reconstruction
Source: Sci Rep. 2023 Apr 4;13:5496. doi: 10.1038/s41598-023-30245-3 (PMC10071462; doi:10.1038/s41598-023-30245-3)

Expanded forehead flap in Asian nasal reconstruction

Muqian Wei#, Xi Bu#, Guanhuier Wang#, Yonghuan Zhen, Xin Yang, Dong Li, Yang An*

1. Department of Plastic Surgery, Peking University Third Hospital, 49 North Garden Road, Haidian District, Beijing, 100191, China

#These authors contributed equally to this work

*Corresponding author:

Yang An MD.

Email: anyangdoctor@163.com;

Supplementary Table S1. Patients Characteristics

Patient	Age, yr	Sex	Nationality	Smoking	Drinking	Comorbidities	BMI	Case of Nasal Injury	Site of Nasal Injury	Follow-up time (years)
1	31	M	Manchu	No	Yes	-	20.07	Cicatricial contracture	St + C + A + T + D	11
2	31	M	Tibetan	No	No	-	18.40	Trauma	All nasal tissue below keystone (including cartilage and soft tissue)	8
3	45	M	Han	No	No	-	29.53	SCC	Right A	8
4	31	F	Han	No	No	-	22.03	Mucormycosis	All nasal soft tissue, cartilage and bone	7
5	55	F	Chaoxian	No	No	-	21.23	Secondary contracture	-	9
6	66	F	Han	No	No	-	25.28	SCC	Right A + T	8
7	52	M	Han	Yes (SI=60)	No	-	25.46	SCC	Right A	9
8	28	M	Han	No	No	-	20.07	Trauma	Right A + T	7
9	35	F	Han	No	No	-	17.58	Severe saddle nose	-	6
10	26	F	Han	No	No	-	18.73	Rat bite	A + T	4
11	56	M	Han	No	No	Hypertension, Diabetes, CHD	32.05	Trauma	Left St +Left A	-
12	53	M	Han	No	No	-	23.73	Trauma	Right St + Right T	-
13	70	M	Han	Yes	Yes	Hypertension, CHD	21.97	BCC	Left A + Left Lw	-

Abbreviations: A, ala; BCC, basal cell carcinoma; BMI, body mass index; C, columella; CHD, coronary heart disease; D, dorsum; F, female; Lw, lateral wall; M, male; SCC, squamous cell carcinoma; SI, Smoking Index; St, soft triangle; T, tip.

Supplementary Figure S1. Patient 4 with a total nasal defect.

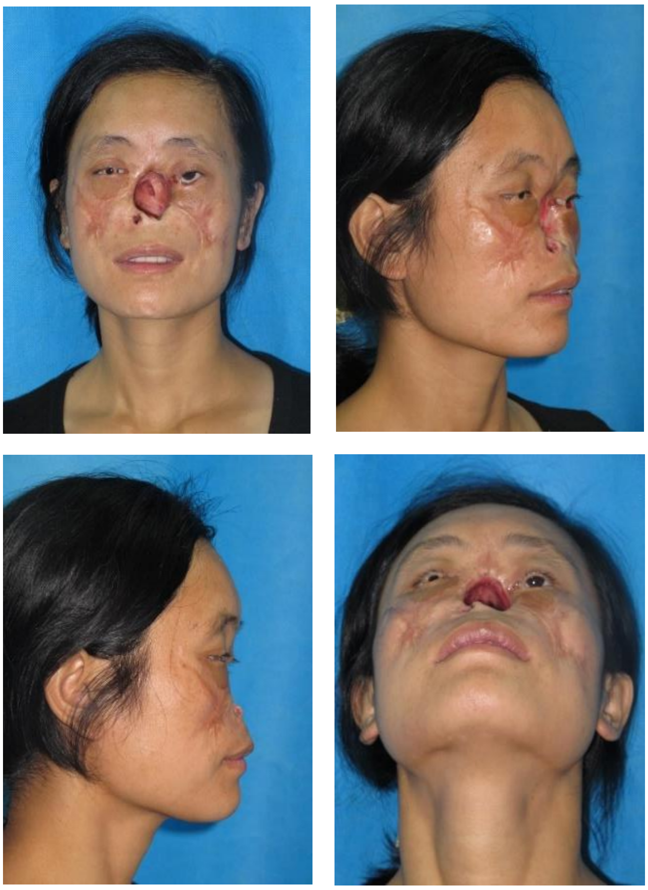

Supplementary Figure S2. Design of the treatment plan. (A) Defect of the nose. (B) Implantation of tissue expander. (C) Transfer of the radial forearm free flap and great saphenous vein. (D) Nasal reconstruction with expanded forehead flap, auricular and costal cartilage, and rib. (E) Dissection of the pedicle. (F) Final result of nasal reconstruction.

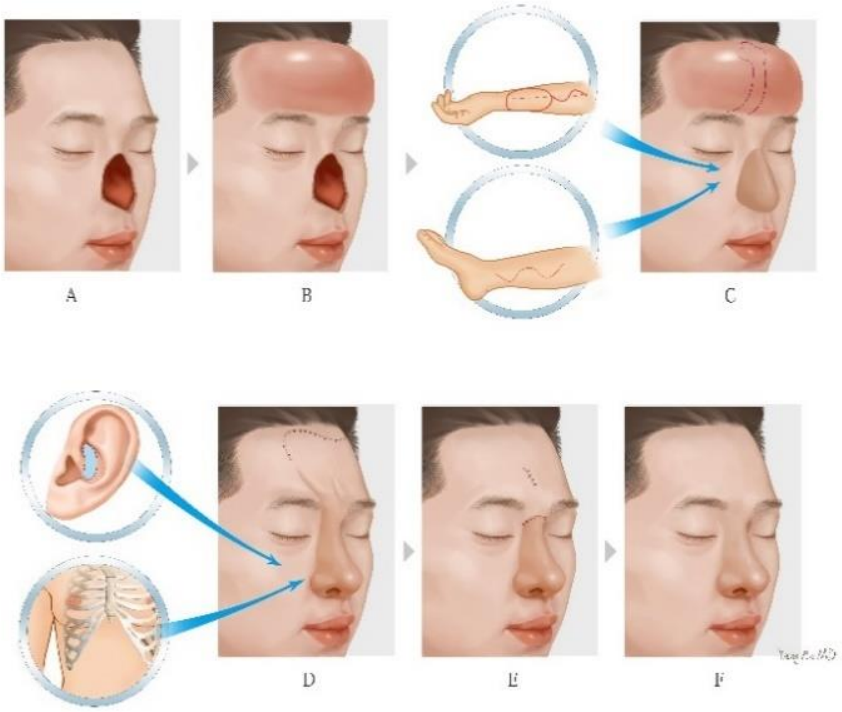

Supplementary Figure S3. Implantation of the tissue expander.

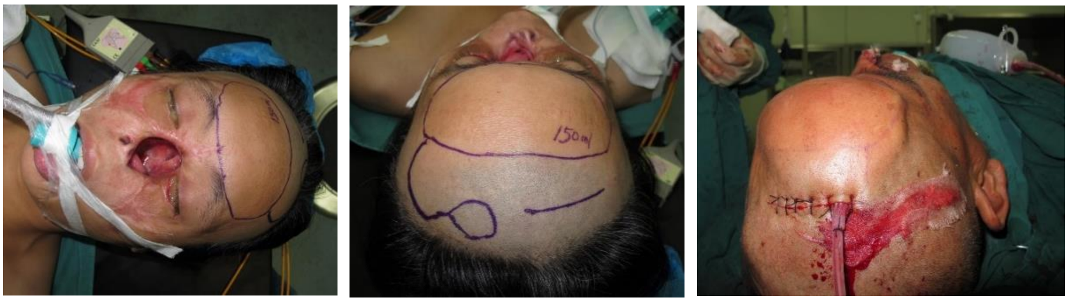

Supplementary Figure S4. A 400 ml expansion volume.

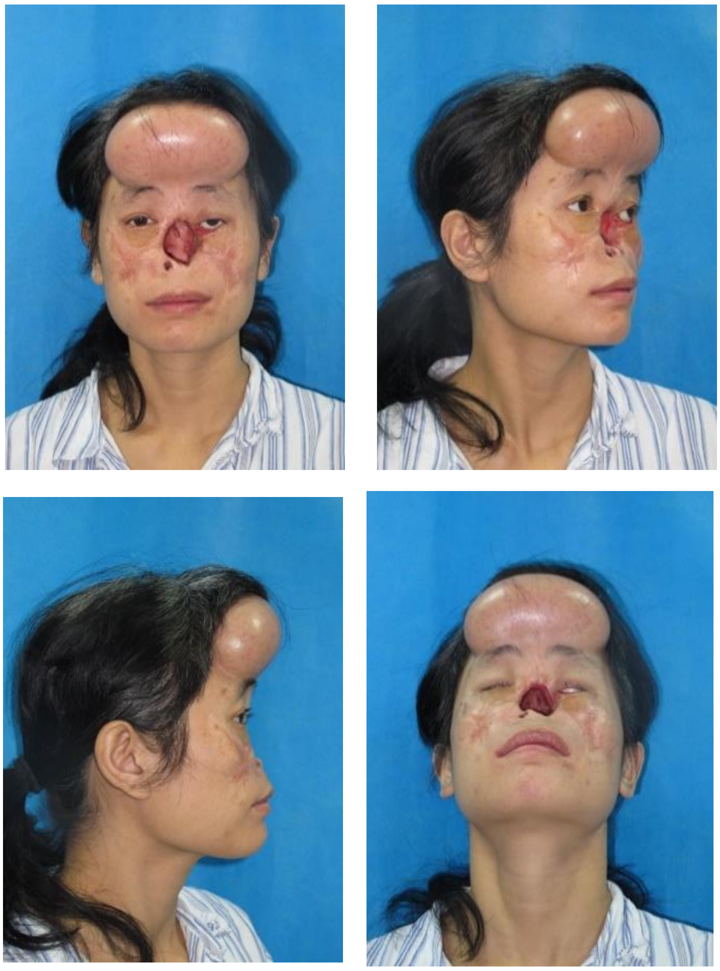

Supplementary Figure S5. (A) A left radial forearm free flap (11cmx7.5cm). (B) A 12cm long right great saphenous vein. (C, D) The flap was transferred to fill the nasal cavity.

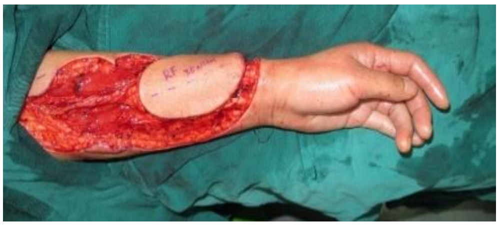

A

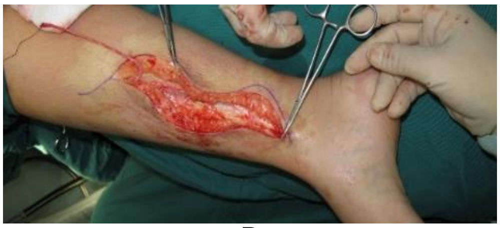

B

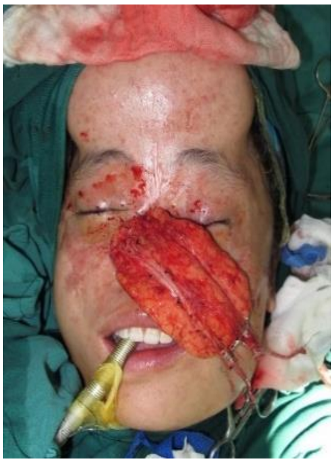

C

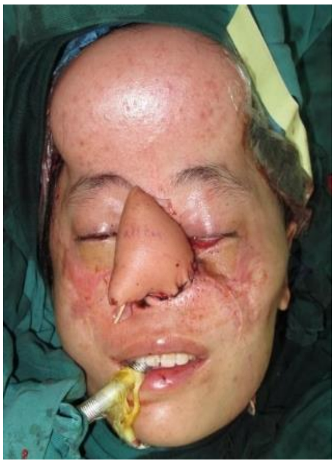

D

Supplementary Figure S6. The right 7th rib and costal cartilage.

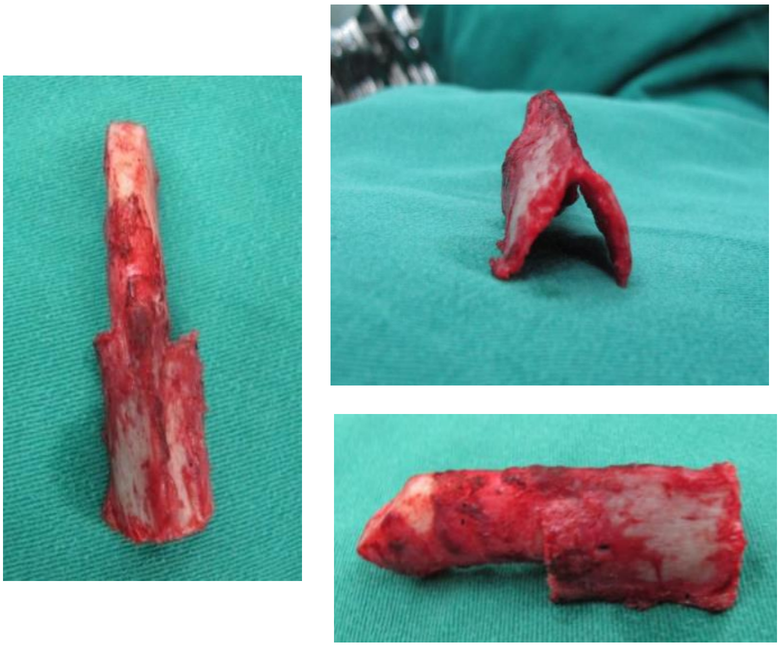

Supplementary Figure S7. Total nasal reconstruction with expanded paramedian forehead flap.

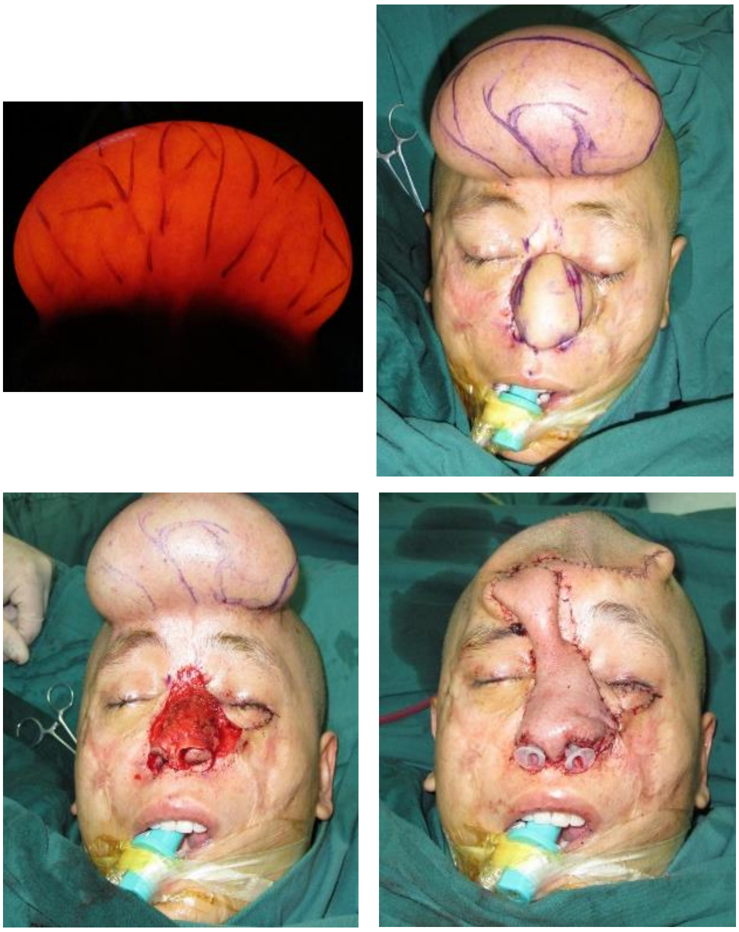

Supplementary Figure S8. Pedicle dissection of the flap.

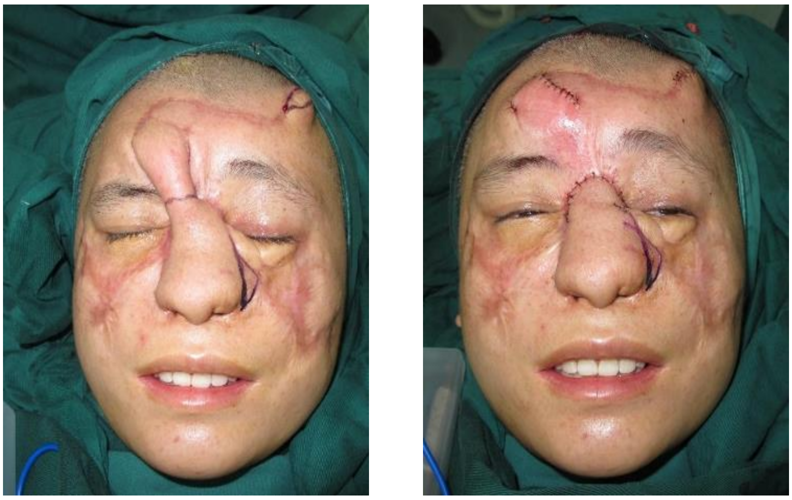

Supplementary Figure S9. 1-year follow-up postoperative result of patient 4.

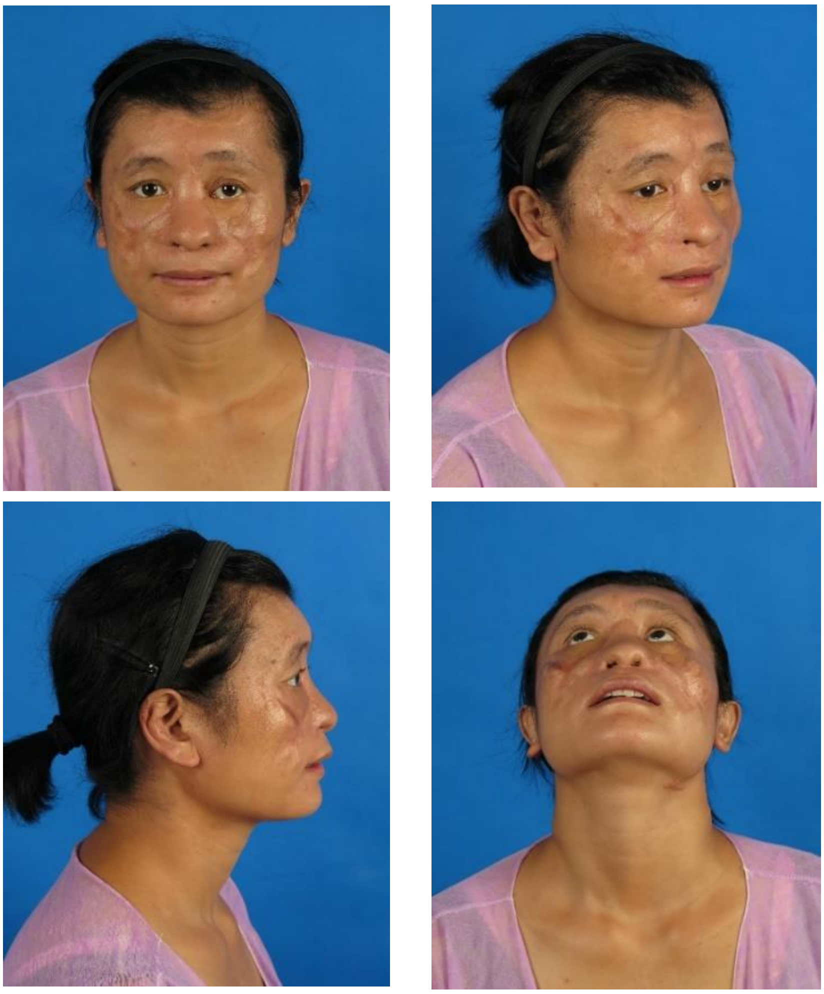

Supplement: Supplementary file 1 — Supplementary Information. [file 41598_2023_30245_MOESM1_ESM.pdf]
